# Anti-tumor effects of tirbanibulin in squamous cell carcinoma cells are mediated via disruption of tubulin-polymerization

**DOI:** 10.1007/s00403-024-03032-x

**Published:** 2024-06-07

**Authors:** Viola K. DeTemple, Antje Walter, Sabine Bredemeier, Ralf Gutzmer, Katrin Schaper-Gerhardt

**Affiliations:** 1 Universitätsklinik für Dermatologie, Venerologie, Allergologie und Phlebologie, Johannes Wesling Klinikum Minden, Universitätsklinik der Ruhr-Universität Bochum, Hans-Nolte-Straße 1, 32429 Minden, Germany; 2 Klinik für Dermatologie, Allergologie und Venerologie, Hauttumorzentrum Hannover, Medizinische Hochschule Hannover, Carl-Neuberg-Str. 1, 30625 Hannover, Germany

**Keywords:** Cutaneous squamous cell carcinoma (cSCC), Actinic keratosis (AK), Tirbanibulin,, Beta-tubulin-polymerization

## Abstract

Topical tirbanibulin is a highly effective and well tolerated novel treatment option for actinic keratoses (AKs). This study aimed to characterize the mode of action of tirbanibulin in keratinocytes (NHEK) and cutaneous squamous cell carcinoma (cSCC) cell lines (A431, SCC-12) in vitro. Tirbanibulin significantly reduced proliferation in a dose-dependent manner in all investigated cell lines, inhibited migration, and induced G2/M-cell cycle arrest only in the cSCC cell lines analyzed, and induced apoptosis solely in A431, which showed the highest sensitivity to tirbanibulin. In general, we detected low basal expression of phosphorylated SRC in all cell lines analyzed, therefore, interference with SRC signaling does not appear to be the driving force regarding the observed effects of tirbanibulin. The most prominent tirbanibulin-mediated effect was on β-tubulin-polymerization, which was especially impaired in A431. Additionally, tirbanibulin induced an increase of the proinflammatory cytokines IL-1α, bFGF and VEGF in A431. In conclusion, tirbanibulin mediated anti-tumor effects predominantly in A431, while healthy keratinocytes and more dedifferentiated SCC-12 were less influenced. These effects of tirbanibulin are most likely mediated via dysregulation of β-tubulin-polymerization and may be supported by proinflammatory aspects.

## Introduction

Actinic keratoses (AKs) are the most frequent precancerous skin lesions characterized by intradermal hyperproliferation of atypical keratinocytes [1], presenting as erythematous irregular or sharply defined macules or plaques with increased keratosis and scaling [2], which spread from a few millimeters up to field cancerization. The most important risk factor for development of AKs is chronic sun exposure leading to occurrence especially on the face, scalp, arms, and legs. Other risk factors include advanced age, male sex, skin type I-II, and chronic immunosuppression [3]. Up to 20% of AKs per lesion and year progress to a cutaneous squamous cell carcinoma (cSCC) [1, 4, 5]. Thus, with a constantly increasing incidence of currently 70–80% in adults older than 60 years [3], AKs pose a relevant clinical issue.

To meet this challenge, treatment options – depending on patient-, lesion- and therapy-specific factors [6] – include cryosurgery, photodynamic therapies, laser procedures and topical agents [2, 6, 7]. Due to commonly long topical treatment intervals from 1–6 months with applications once or twice daily [8], and/or adverse events (AEs) such as pruritus or erythema, seldomly also scarring, necrosis or angioedema [9], compliance is often insufficient leading to suboptimal treatment success.

In 2021, the food and drug administration approved tirbanibulin 1% ointment as a novel topical treatment option for AKs. In two phase 3 clinical trials, after a single 5 day treatment cycle, 4–49% of patients presented with 100% clearance of AKs within 8–9 weeks. About 16% of patients experienced mild AEs, e.g., erythema, scaling, pruritus, or pain at the application site. Recurrence was observed in 57% of patients after 12 months [10].

In different cancer models, e.g., breast and ovarian cancer, tirbanibulin (KX-01 or KX2-391) acted as inhibitor of tubulin-polymerization and of the oncogenic SRC-kinase [11–13], thereby inducing p53 and G2/M-cell cycle arrest, which subsequently lead to apoptosis by stimulating caspase 3 and poly-adenosine diphosphate-ribose [14].

Since the mode of action in keratinocytes and keratinocyte-derived carcinoma cells is unclear, we analyzed effects from tirbanibulin on normal human epithelial keratinocytes (NHEK) and cSCC cells in vitro.

## Materials and methods

### Reagents

Tirbanibulin (Selleck Chemicals LLC, Houston, TX, U.S.) was dissolved in DMSO (Merck, Darmstadt, Germany).

### Cell culture

Neonatal Normal Human Epidermal Keratinocytes (Lonza, Walkersville, MD, U.S.) were cultured in Basal Medium 2 (PromoCell, Heidelberg, Germany) supplemented with Keratinocyte Growth Medium 2 Supplement Pack (PromoCell).

The A431 cell line (human cutaneous epidermoid carcinoma, female origin, RRID:CVCL_0037) was obtained from Cell Lines Services (Eppelheim, Germany) and cultured in Dulbecco’s Modified Eagle’s Medium with 4.5 g/l Glucose and L-Glutamine supplemented with 10% fetal calf serum (PAN-Biotech, Aidenbach, Germany).

The human cSCC cell line SCC-12 was kindly provided by Prof. Jens Malte Baron from Aachen, Germany (male origin, RRID:CVCL_4026). Cells were cultured in Ham´s F-2 Medium (Biochrom, Berlin, Germany) and Dulbecco’s Modified Eagle’s Medium (PAN-Biotech) in a 1:4-ratio, supplemented with 10% fetal calf serum, 1% Penicillin/Streptavidin (Biochrom), 40 ng/ml Hydrocortisone (Sigma-Aldrich, Darmstadt, Germany), 10 ng/ml EGF (Roche, Basel, Schweiz) and 5 µg/ml Insulin (PromoCell).

Cells were incubated at 37 °C in a humidified 5% carbon dioxide atmosphere.

The cell lines used differ in sex of donors. Since we did not analyze sex-specific differences, this can be viewed as a limitation concerning our analyses.

### 3-(4,5-dimethylthiazol-2-yl)-2,5-diphenyltetrazolium bromide (MTT) assay

Cells were plated in 96-well flat-bottom plates (Sarstedt, Nümbrecht, Germany) and treated with tirbanibulin (0 nM−100 nM). Six replicates were performed per condition. Proliferation was evaluated via Cell Titer 96^®^ Non-radioactive Cell Proliferation Assay (Promega, Maddison, WI USA), absorption measured with Fluostar (BMG Labtechnologies, Ortenberg, Germany).

### Scratch assay

Cells were grown in 6-well plates until reaching confluence. A vertical scratch was set with a 1000 µl pipette tip. Cells were incubated with medium containing (i) DMSO (unstimulated control), (ii) tirbanibulin 50 nM, or (iii) tirbanibulin 100 nM for 24 h at 37 °C. Photo documentation in phase contrast mode on All-in-One Fluorescence Microscope BZ-X800 (Keyence, Osaka, Japan, RRID:SCR_023617) before and after stimulation of the identical area per well. The scratch area was measured using the Keyence Analyzer Software (Keyence). Six replicates were performed per condition and cell line.

### Apoptosis analysis

Cells were cultivated in 6-well plates until reaching confluence and incubated with medium containing (i) DMSO (unstimulated control), (ii) tirbanibulin 50 nM, or (iii) tirbanibulin 100 nM for 24 h (A431) or 48 h (NHEK, SCC-12). Cells from three wells per condition were pooled for further processing and washed in cell staining buffer (BioLegend, San Diego, CA, U.S.) before resuspension in Annexin V binding buffer (BioLegend). Staining with 7AAD (BioLegend) and FITC Annexin V (BioLegend) followed by incubation for 20 min at RT. Prior to measurement on the flow cytometer (Cytoflex, Beckman Coulter, Brea, CA, U.S.), cell suspension was diluted with Annexin V binding buffer. 10.000 events were recorded per condition. Six replicates were performed per condition and cell line.

### Western blot

200.000 cells were cultivated in 6-well plates and stimulated with different concentrations of tirbanibulin (20–80 nM) for 5–30 min. Cells were incubated with Pierce^™^ RIPA buffer (Thermo Scientific, Rockford, IL USA) supplemented with 0.1% Halt^™^ Protease & Phosphatase Inhibitor Cocktail (Thermo Scientific) on ice for 15 min under agitation before cell collection and centrifugation for 15 min at 14,000×*g*. Protein concentration was determined with Lowry Protein Assay (BioRad, Buckinghamshire, UK) according to the manufacturer’s instructions. Absorbance was measured on Fluostar.

After electrophoresis, dispersed proteins were blotted onto a nitrocellulose membrane (GE Healthcare, Buckinghamshire, UK) and conjugated with antibodies (all from Cell Signaling, Danvers, MA, U.S.) for SRC (Cat# 2109, RRID: AB_2106059), pSRC (Cat# 6943, RRID: AB_10013641), FAK (Cat# 3285S, RRID: AB_2269034), pFAK (Cat# 8556S, RRID: AB_10891442), ERK (Cat# 4695S, RRID: AB_390779), pERK (Cat# 4370, RRID: AB_2315112), AKT (Cat# 4691S, RRID: AB_915783), pAKT (Cat# 4060S, RRID: AB_2315049), STAT3 (Cat# 12640, RRID: AB_2629499), pSTAT3 (Cat# 9145, RRID: AB_2491009), and GAPDH (loading control, Cat# 2118, RRID: AB_561053). As marker, SuperSignal^®^ Molecular Weight Protein Ladder (Thermo Scientific) was used. Target antibodies were labelled with anti-Rabbit IgG HRP-linked (Cell Signaling, Cat# 7074S, RRID: AB_2099233) and detected with Lumi Light PLUS Western Blotting Substrate (Thermo Scientific) using an Aequonia Multifunction Darkbox System (Hamamatsu, Hamamatsu City, Japan).

### Human phosphokinase-assay

Phosphorylation screening via Proteome Profiler^™^ Array was performed using the Human Phospho-Kinase Array Kit (R&D Systems, Wiesbaden, Germany) according to the manufacturer’s instructions.

### Electrochemiluminescence-assay

Cells were stimulated with different concentrations of tirbanibulin (20 nM, 50 nM). Supernatants were collected after 24 h of incubation at 37 °C. Immunoassay (V-Plex-54, U-Plex) according to the manufacturer’s instructions. Measurement on MESO QuickPlex SQ120. Quantification using Mesoscale Discovery Workbench 4.0 software (Mesoscale Discovery, MD, U.S.).

### Enzyme-linked immunosorbent assay (ELISA)

IL-1α, bFGF and Flt-1 were measured in the supernatants from cells stimulated with tirbanibulin (50 nM/100 nM) by ELISA using commercially available kits according to the manufacturer’s protocol (R&D Systems). Absorbance was measured on Spectra Max iD3.

### Cell cycle analysis

Cells were stimulated in 6-well plates after reaching confluence with medium containing (i) DMSO (unstimulated control), (ii) tirbanibulin 50 nM or (iii) tirbanibulin 100 nM for 24 h (A431) or 48 h (NHEK, SCC-12). Cells from three wells per condition were pooled and fixed with ice-cold ethanol 70% (Sulpeco, Sigma-Aldrich) for at least 30 min. After staining with 7AAD for 20 min at RT, cells were measured on the flow cytometer. 25.000 events were recorded per condition. Six replicates were performed per condition and cell line.

### β-tubulin polymerization-assay

Cells were cultivated in 6-well plates until reaching confluence. Incubation with medium containing (i) DMSO (unstimulated control), or (ii) tirbanibulin 50 nM for 24 h (A431) or 48h (NHEK, SCC-12). β-tubulin-polymerization analysis using the Microtubules / Tubulin In Vivo Assay Kit (Cytoskeleton, Inc., Denver, CO, U.S.). The protocol was modified as follows: 100 µl lysed cells were processed per condition. Following centrifugation at 1000×*g* for 5 min at 37 °C, low speed supernatants were centrifuged at 20.630×*g* for 90 min at 37 °C. 80 µl of high-speed supernatants (HSS) were collected and 20 µl 4× Laemmli Buffer (BioRad) was added. Low speed pellets (LSP) were resuspended in 120 µl 2× Laemmli Buffer (diluted with Ampuwa). HSS and LSP were stored at – 20 °C. After thawing samples at 95 °C for 5 min, gel electrophoresis was performed with Mini-Protean TGX 4–20% precast gels (BioRad) or Mini-Protean TGX 10% precast gels (BioRad) using a Mini-PROTEAN Tetra Cell (BioRad) at 300 V for approximately 15 min. Tubulin Protein Standard (provided in the kit) was diluted as described by the manufacturer with 4× Laemmli Buffer. For blotting, Trans-Blot Turbo Transfer Packs (mini format, 0.2 µm PVDF, BioRad) were used on the Trans-Blot^®^ Turbo^™^ Transfer System (BioRad). Blotting at 1.3 A for 7 min prior to blocking with EveryBlot Blocking Buffer (BioRad) for 30min at RT. Following a washing step with TBS (BioRad) supplemented with Tween (Sigma-Aldrich) (TBS-T), the primary antibody (anti-Tubulin, sheep polyclonal antibody, Cytoskeleton, Cat# ATN02, RRID: AB_10708807) was added in a dilution of 1:1000 in EveryBlot Blocking Buffer. Incubation for 1h at RT. After washing, anti-sheep HRP conjugated secondary antibody (Cytoskeleton) was added in a dilution of 1:10.000 in EveryBlot Blocking Buffer. Incubation for 1h at RT. Following the final washing step, incubation with Clarity Western ECL Substrate mixture (BioRad) for 5 min in the dark at RT. Chemiluminescence measurement on ChemiDoc^™^ Imaging System. Analysis with Image Lab Software. Three replicates were performed per condition and cell line.

### Immunocytochemistry

Cells were cultivated in 24-well plates until reaching a confluence of 10–30%. Incubation with medium containing (i) DMSO (unstimulated control), (ii) tirbanibulin 50 nM, or (iii) tirbanibulin 100 nM for 24 h (A431) or 48 h (NHEK, SCC-12). Fixation with 4% Formalin (Roth, Karlsruhe, Germany) for 10 min at RT. Permeabilization with 0.1% Triton^™^ X-100 (Merck) for 15 min at RT. Blocking with 5% bovine serum albumin (Miltenyi Biotec, Bergisch Gladbach, Germany) in PBS for 45 min at RT. Incubation with the primary antibody (β-tubulin (9F3) Rabbit mAb, Cell Signaling, Cat# 2128S, RRID: AB_823664) in a dilution of 1:200 in PBS over night at 4 °C. Incubation with secondary antibody (anti-rabbit IgG Fab2 Alexa Fluor^®^ 647, Cell Signaling, Cat# 4414 RRID:AB_10693544) in a dilution of 1:1000 in PBS for 1 h at RT in the dark. Nuclear staining using FluoroMounter with DAPI (Bio SB, Santa Barbara, CA, U.S.). Imaging on All-in-One Fluorescence Microscope. Three replicates were performed per condition and cell line.

### Statistical analysis

Statistical analyses with GraphPad Prism (version 5.02, GraphPad Software, Boston, MA, U.S.). Applied tests (nonlinear regression, paired t-test, unpaired t-test, Dunn’s multiple comparisons test) are stated in the respective figure legend. p-values < 0.05 were regarded as statistically significant. Curve fitting and visualization as boxplots performed with GraphPad Prism. Bar charts prepared using Excel Software. Heatmaps created with R Studio (R version 4.2.3, packages ComplexHeatmap, Posit Software, PBC formerly RStudio, PBC, Boston, MA, U.S.).

## Results

### Tirbanibulin inhibits proliferation in NHEK, A431 and SCC-12

**Fig. 1:**
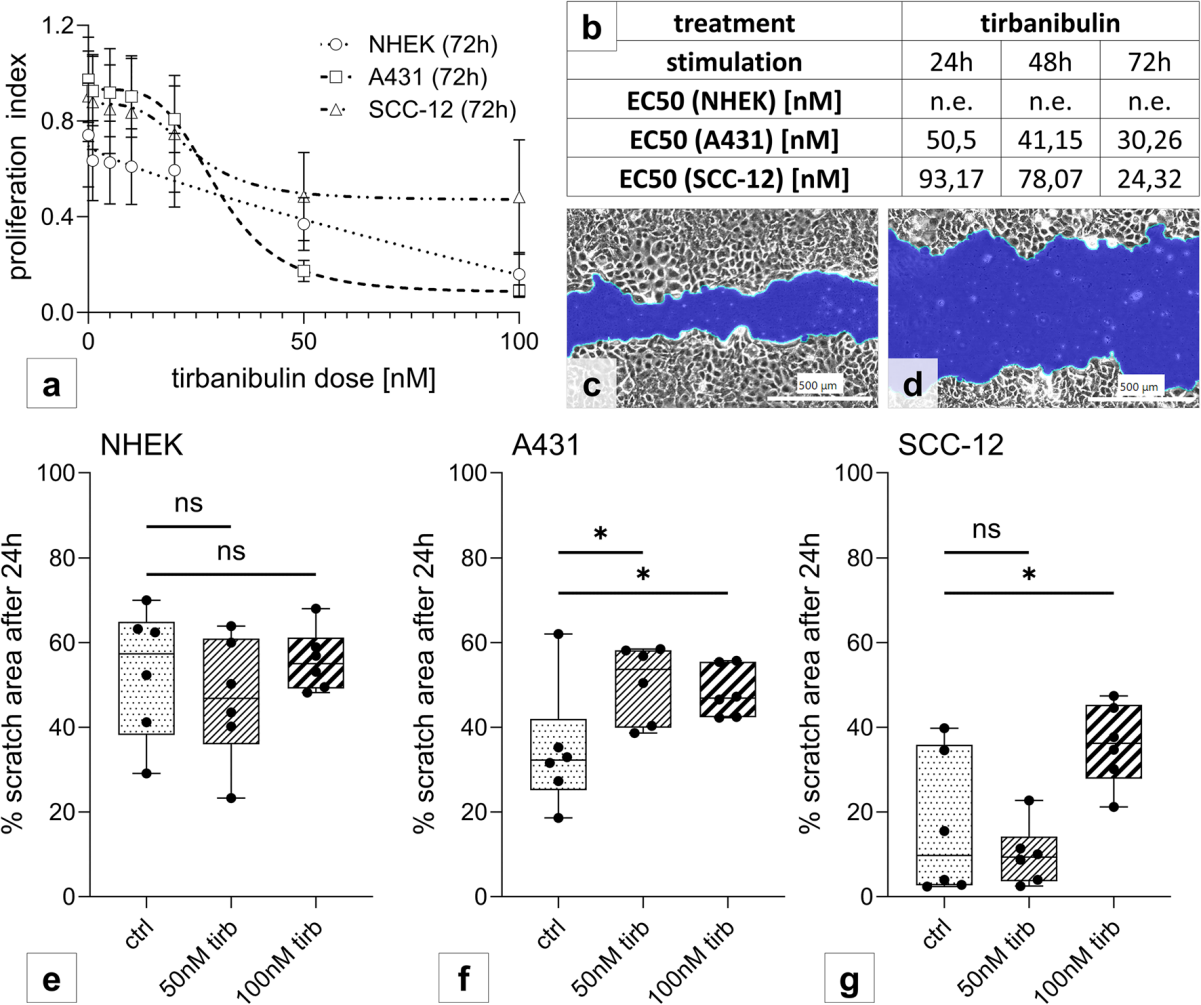
Tirbanibulin inhibits proliferation and migration in keratinocytic cell lines. **a**, **b** Cells were stimulated with tirbanibulin (0 nM–100 nM) over 24–72 h. Proliferation index was measured via MTT-Test. EC50 calculation and curve fitting occurred with nonlinear regression analysis. Data points represent means +/− SD of 3–6 replicates per condition. **c**–**g** Scratch test. Representative photo documentation of scratch area (in blue) in A431 24 h after setting the scratch, **c** unstimulated and **d** with stimulation (50 nM tirbanibulin). Scale bar = 500 µm. Percental difference between measured area at 0 h and 24 h was determined for **e** NHEK, **f** A431 and **g** SCC-12 following stimulation with 0 nM, 50 nM or 100nM tirbanibulin. Pattern according to stimulation condition. Statistical analysis with paired t-test (ns p > 0.05; *p < 0.05). Six replicates were performed per condition

Cell proliferation was measured via MTT-assay. Whilst tirbanibulin-stimulation for 24 h showed no effect, prolonged stimulation (48 h/72 h) proved a significant dose-dependent inhibition of proliferation in A431 and SCC-12, while NHEK presented with a limited effect at the highest drug concentration assessed (100 nM). Notably, proliferation index was reduced by 50% following 72 h of stimulation with tirbanibulin at a concentration of 30.26 nM for A431 and of 24.32 nM for SCC-12 (Fig. 1a, b).

### Tirbanibulin inhibits migration in cSCC cell lines

Migration was evaluated via scratch test. Whilst NHEK were unaffected by tirbanibulin (Fig. 1e), tirbanibulin (50 nM/100 nM) significantly reduced migration in A431 (Fig. 1c, d, f), and in SCC-12 at 100 nM concentration of tirbanibulin (Fig. 1g).

### Tirbanibulin inhibits apoptosis in A431

**Fig. 2:**
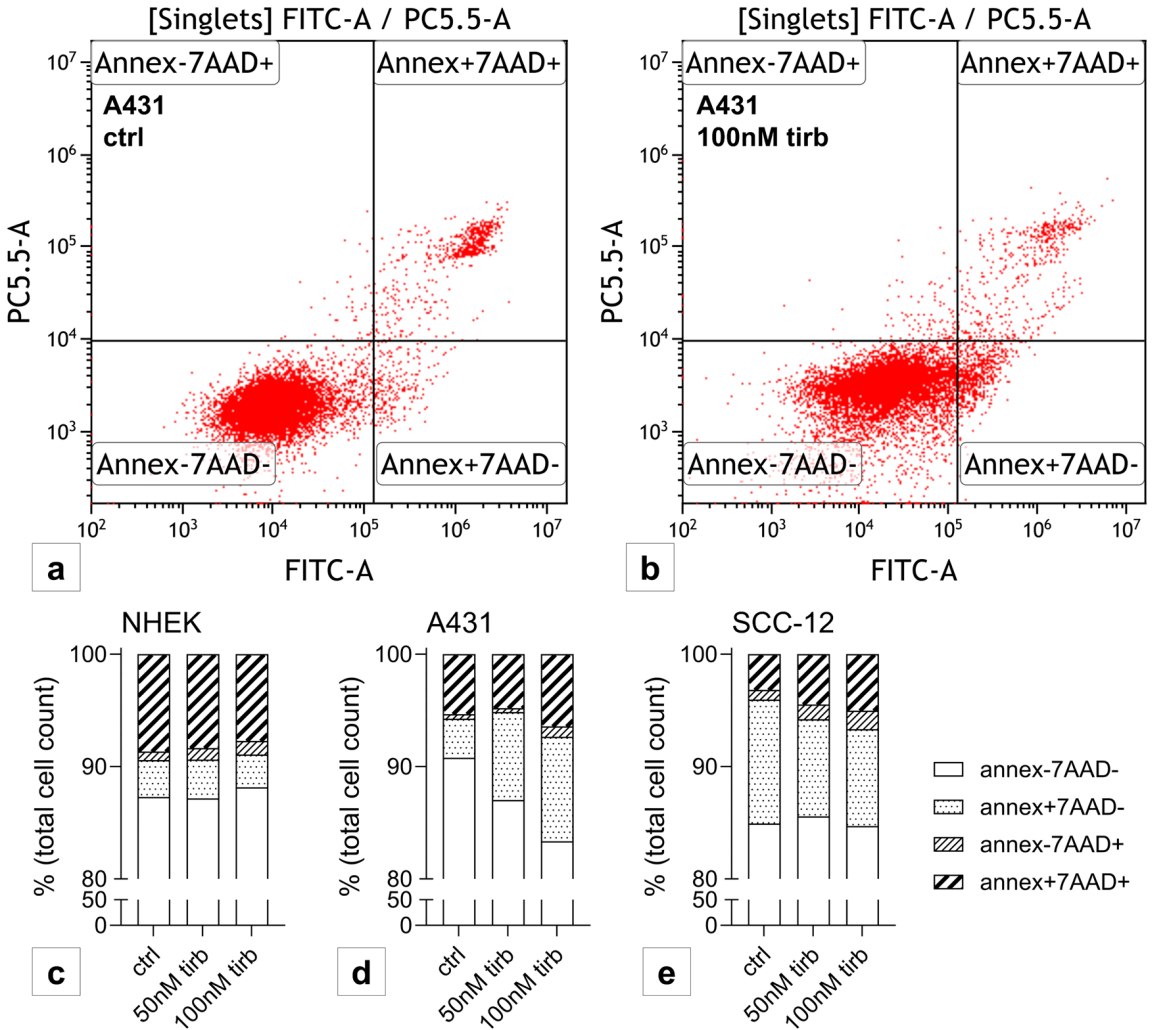
Tirbanibulin induces apoptosis in A431. Flow cytometry measurement after staining with 7AAD and Annexin V. Representative flow cytometry analysis of A431 **a** with DMSO control and **b** stimulated with 100 nM tirbanibulin for 24 h. Bar charts represent percentage of total cell count per staining status stacked by stimulation condition for **c** NHEK, **d** A431 and **e** SCC-12. Pattern according to staining status: Annexin-7AAD- represent live cells, Annexin + 7AAD- apoptotic cells, Annexin-7AAD + necrotic cells, Annexin + 7AAD + dead cells. Statistical analysis with Dunn’s multiple comparisons test (not shown). Six replicates were performed per condition

Apoptosis was analyzed via 7AAD/annexin V staining. Again, there was no effect in NHEK (Fig. 2c), but also none in the dedifferentiated SCC-12 (Fig. 2e). Induced apoptosis was seen only in A431 (Fig. 2a, b, d). The increase of necrotic cells was statistically significant in A431 (Fig. 2d) as well as the increase of dead cells in SCC-12 (Fig. 2e).

### No significant impact of tirbanibulin on SRC-signaling in NHEK and cSCC cells

**Fig. 3:**
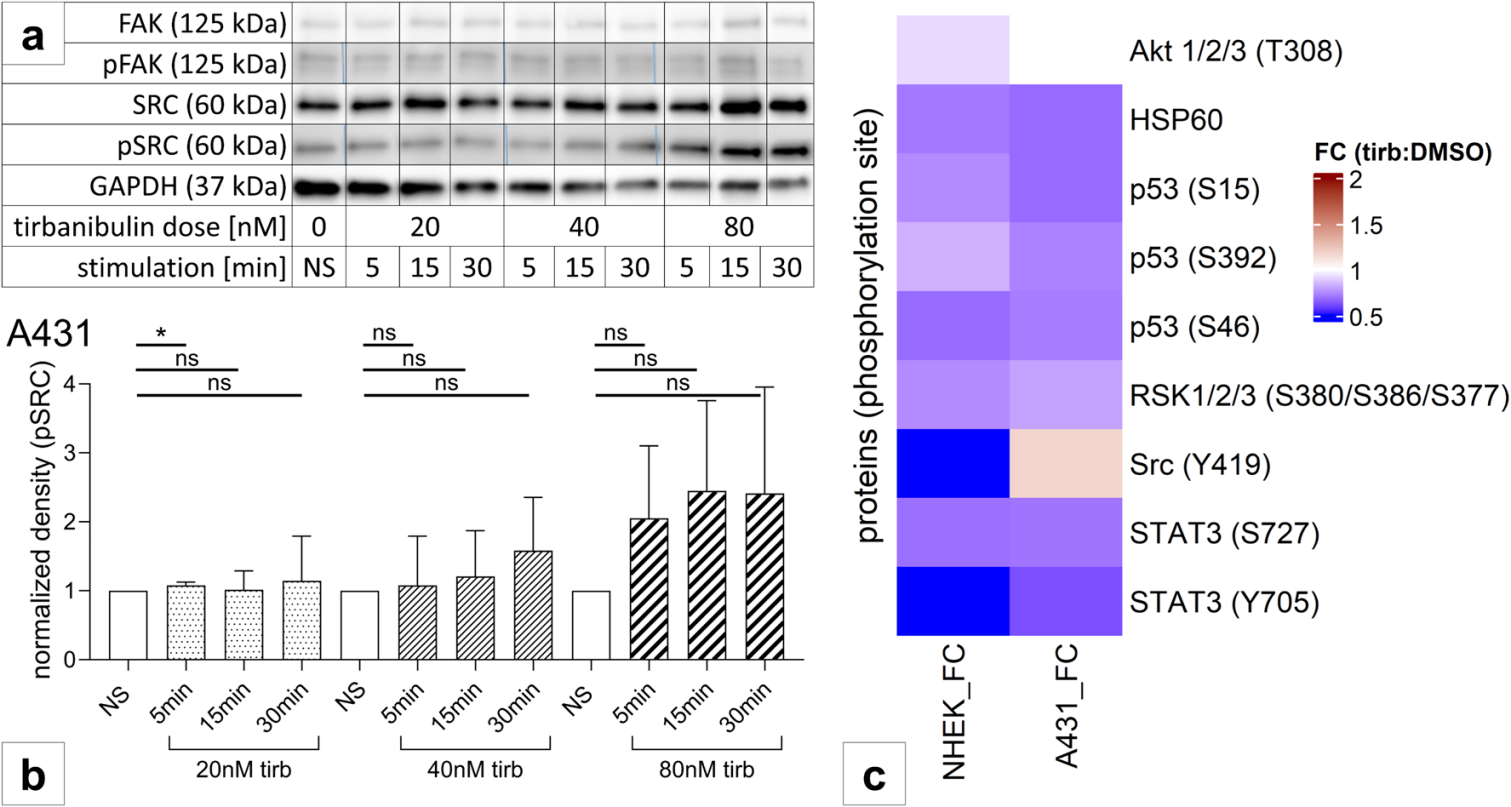
Tirbanibulin induces protein phosphorylation. **a** Representative western blot in A431 after stimulation with 20 nM, 40 nM or 80 nM tirbanibulin for the indicated time intervals. GAPDH served as reference protein. **b** Phosphorylation of SRC depending on dose and stimulation duration in A431. Bar charts depict means +/− SD for three replicates per condition. Pattern according to stimulation condition. Statistical analysis with unpaired t-test (ns p > 0.05; *p < 0.05). **c** Phosphokinase array on NHEK and A431. Ratio between the volume (density) of target protein dots and of the integrated reference spot was measured (data not shown), heat map of fold change (FC) comparing tirbanibulin stimulated sample with DMSO control sample

Looking into the molecular mechanisms behind the observations described, we examined the effects of tirbanibulin on SRC-signaling. We first assessed the baseline expression of phosphorylated SRC (pSRC) by western blot. Here, pSRC-expression was very low, with no further reduction after tirbanibulin treatment, whereas non-phosphorylated SRC was detected both before and after tirbanibulin treatment (Fig. 3a). Also, SRC downstream signaling (represented by FAK, ERK, AKT and STAT3) was not altered in NHEK and SCC-12 following tirbanibulin treatment (data not shown). In A431, however, we observed an increased SRC phosphorylation following short-term high dose (80 nM) tirbanibulin stimulation (Fig. 3a, b). Screening for 37 kinase phosphorylation sites (Fig. 3c), relevant reduction of phosphorylation via tirbanibulin (fold change < 0.5 between stimulated sample and unstimulated control) was seen for SRC (Y419) and STAT3 (Y705) in NHEK. Tendencies of reduced phosphorylation (fold change < 0.75) was seen for HSP60 and RSK 1/2/3 (S15/S392/S46) in NHEK and A431, and for STAT3 (S727/Y705) in A431. In A431, in line with our western blot results, there was a tendency of induced phosphorylation for SRC (Y419) following high dose tirbanibulin treatment. This may be regarded as a normalization of pSRC-expression, however, must be treated with caution, since high tirbanibulin concentrations lead to cytotoxicity.

### Tirbanibulin impairs β-tubulin-polymerization in A431

**Fig. 4:**
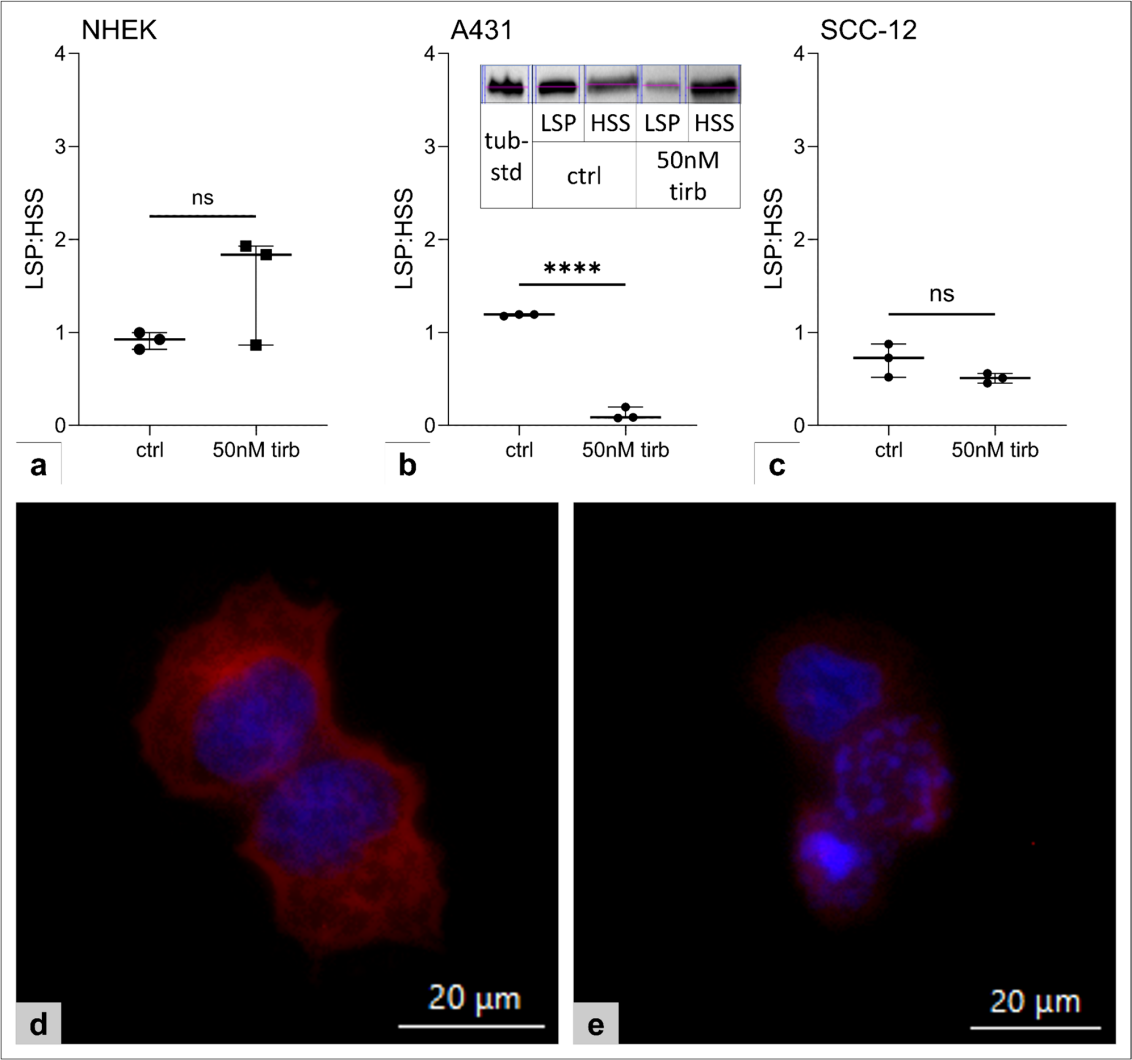
Tirbanibulin prevents β-tubulin-polymerization in A431. **a**–**c** β-tubulin-polymerization was evaluated via LSP:HSS ratio for **a** NHEK, **b** A431 and **c** SCC-12. LSP = low speed pellet, representing stabilized microtubules, HSS = high-speed supernatant, representing free β-tubulin. Representative western blot of A431 after stimulation with tirbanibulin 50 nM for 24 h **b**, inlet. Statistical analysis with unpaired t-test (ns p > 0.05; ****p < 0.0001). Three replicates were performed per condition. **d**, **e** Immunocytochemistry staining of β-tubulin in A431 **d** unstimulated and **e** with stimulation with 50 nM tirbanibulin for 24 h. Scale bar = 20 µm

Alongside SRC-signaling, we also examined ß-tubulin-polymerization. This was significantly inhibited by tirbanibulin in A431 (Fig. 4b); again, NHEK (Fig. 4a) and SCC-12 (Fig. 4c) were unaffected by tirbanibulin, even following prolonged stimulation (48 h). Visualization of β-tubulin was performed via immunocytochemistry: while in control samples a distinct microtubule network was detectable (Fig. 4d), this was shattered in A431 after tirbanibulin stimulation (Fig. 4e). In addition, nuclei presented enlarged and fragmented representing the above-described cell cycle arrest. These effects were less prominent in SCC-12 and lacked in NHEK.

### Tirbanibulin induces cell cycle arrest in A431

**Fig. 5:**
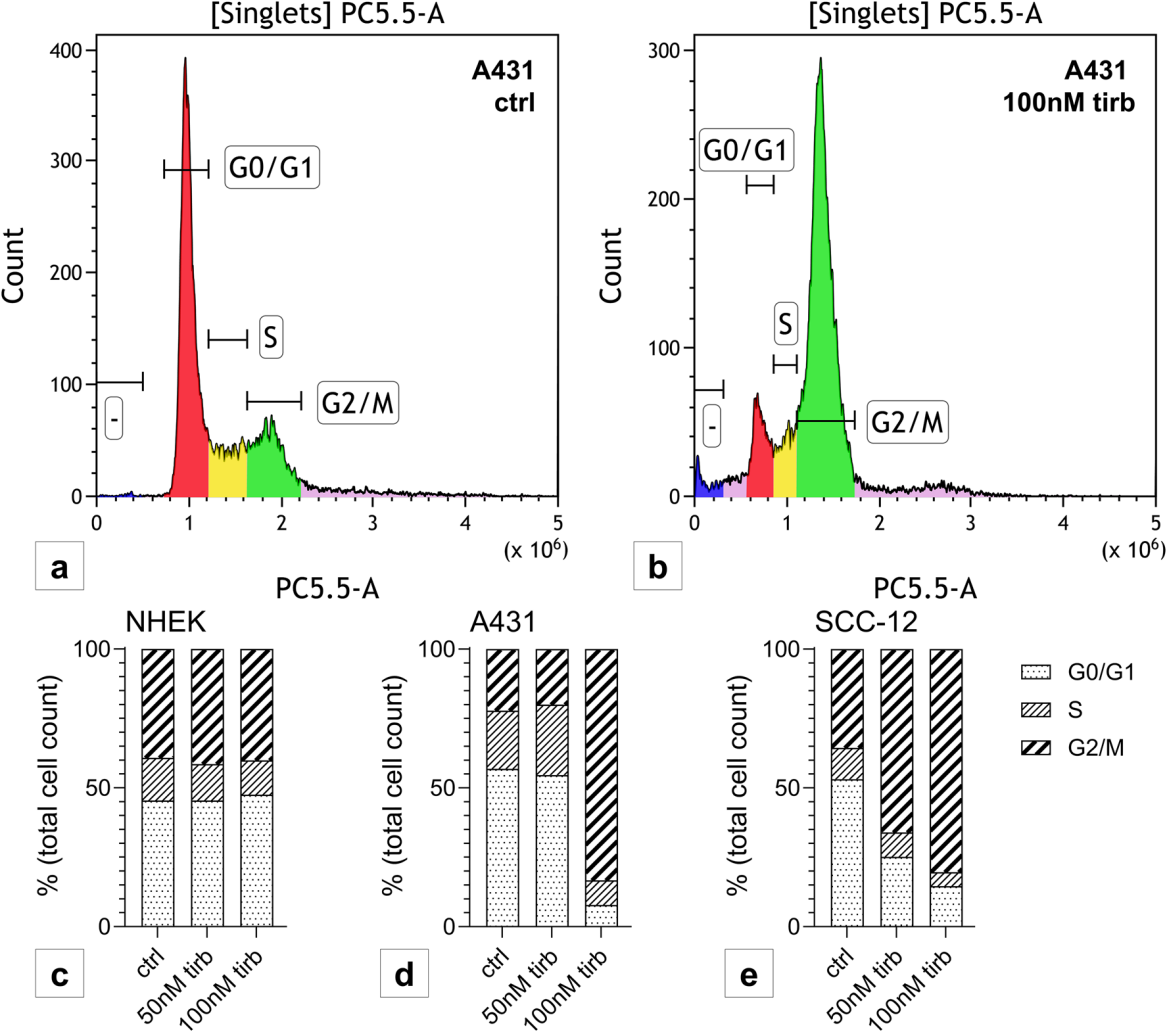
Tirbanibulin induces G2/M-cell cycle arrest in cSCC cells. Representative flow cytometry measurement of A431 after staining with 7AAD, **a** unstimulated, and **b** following stimulation with 100 nM tirbanibulin for 24 h. **c**–**e** Bars represent percentage of total cell count per staining status stacked by stimulation condition for **c** NHEK (48 h), **d** A431 (24 h) and **e** SCC-12 (48 h). Pattern according to cell cycle stage. Statistical analysis with Dunn’s multiple comparisons test (data not shown). Six replicates were performed per condition

Cell cycle progression was analyzed via 7AAD staining. In line with β-tubulin-polymerization, in NHEK, even after 48 h of stimulation, no effect on cell cycle progression was detected (Fig. 5c). This was different in both cSCC cell lines: while tirbanibulin lead to a G2/M-cell cycle arrest in SCC-12 after tirbanibulin stimulation for 48 h (Fig. 5e), this effect was more pronounced in A431 already after 24 h of stimulation (Fig. 5a, b, d).

### Tirbanibulin modulates cytokine secretion

**Fig. 6:**
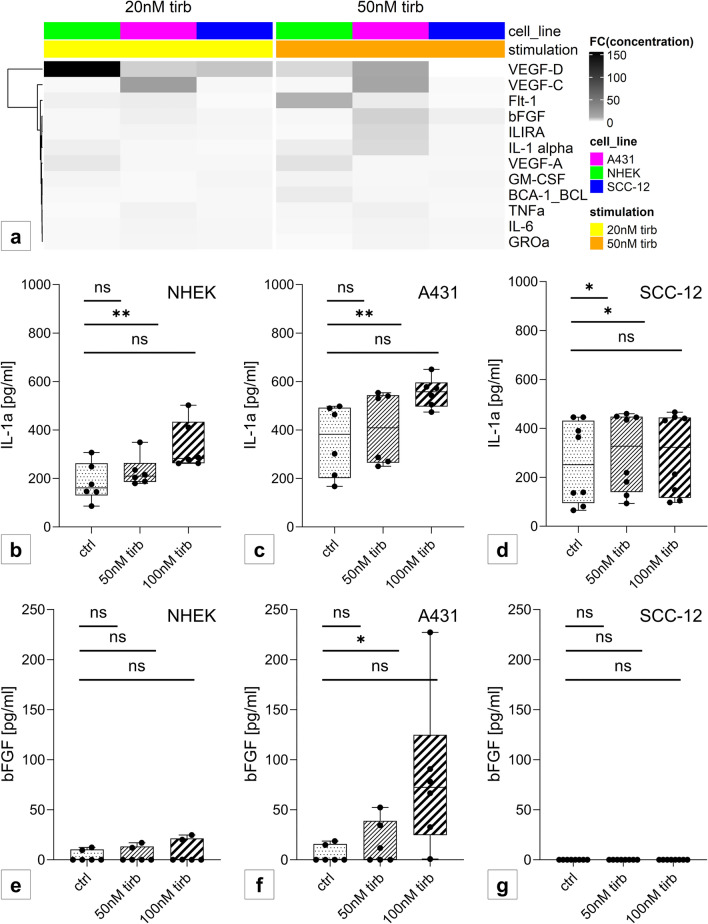
Tirbanibulin alters secretion of multiple VEGF subtypes as well as IL-1α, IL1RA and bFGF. **a** Heat map showing relevant cytokines and chemokines altered by tirbanibulin in NHEK (green), A431 (pink) or SCC-12 (blue). Separation according to stimulation type. Heat represents fold change (ratio stimulated: unstimulated). **b**–**g** Validation via ELISA of cell culture supernatants concerning **b**–**d** IL-1α and **e**–**g** bFGF after stimulation of **b**, **e** NHEK, **c**, **f** A431 and **d**, **g** SCC-12 with 50 nM and 100 nM tirbanibulin for 24 h. Pattern according to stimulation condition. Statistical analysis with paired t-test (ns p > 0.05; *p < 0.05; **p < 0.01). Six replicates were performed per condition

Supernatants of all three cell lines following short-term stimulation with tirbanibulin were analyzed for cytokine and chemokine secretion using a chemiluminescence-screening assay. Fold changes between stimulated samples and unstimulated controls were calculated. Increased secretion was especially detected for VEGF-C and VEGF-D, and to a lesser extent for Flt-1, IL-1α and bFGF. While VEGF-C was only secreted by A431, elevated VEGF-D secretion was additionally detected for NHEK and SCC-12 (Fig. 6a). In a verification enzyme-linked immunosorbent assay on supernatants of further experiments on NHEK, A431 and SCC-12 regarding Flt-1, IL-1α and bFGF, the increase of IL-1α secretion in NHEK (Fig. 6b) and A431 (Fig. 6c), as well as increase of bFGF in A431 (Fig. 6f) were confirmed. Flt-1 was not detected in either cell line (data not shown).

## Discussion

To date, effects of tirbanibulin were analyzed in tumor entities such as breast cancer [11] and mucinous ovarian carcinoma [12]. In an orthotopic mouse model for the latter, tirbanibulin – orally applied—reduced tumor weight and frequency. This in vivo effect was ascribed to inhibited proliferation, and increased apoptosis. Further in vitro analyses on mucinous ovarian carcinoma cell lines revealed a decreased survival and proliferation upon treatment with tirbanibulin, in addition to G2/M-cell cycle arrest and reduced migration [12]. However, there are no data on effects of tirbanibulin on keratinocytic cells, while clinical studies demonstrated a positive effect of tirbanibulin ointment in AKs. Here, we show for the first time that tirbanibulin acts on keratinocytic cell lines by inhibition of proliferation in all three investigated cell types, while reduced migration was apparent only in cSCC cell lines. Apoptosis and cell cycle arrest was solely induced in A431.

These tirbanibulin-mediated effects have previously been explained by the combined inhibition of (i) the SRC tyrosine kinase pathway, and (ii) tubulin-polymerization [12]. Therefore, we analyzed these mechanisms on our keratinocytic cells.

SRC, a non-receptor tyrosine kinase, is an oncogene activated in epithelial tumor entities such as colon and breast cancer [15]. Its phosphorylated form (pSRC) supports proliferation, survival, migration, and angiogenesis thus endorsing cancer progression. Previously, SRC-expression was detected in basal keratinocytes to participate in regulation of physiological cell proliferation and differentiation, and ubiquitously in AKs and cSCC [16]. Additionally, sustained SRC over-expression can induce cellular transformation with the potential to induce hyperproliferative keratinocytic disorders, e.g., psoriasis, but also malignant skin tumors including cSCC [15, 17]. Concordantly, we detected unphosphorylated SRC across all keratinocytic cell lines; in the same unstimulated samples pSRC was present at lower expression levels. The above-mentioned literature displays either SRC or pSRC, respectively, in epithelial skin cancer without comparing the two activation stages. A semi-quantitative comparison via western blot was, however, done in breast cancer cell lines, revealing similar amounts of pSRC and SRC in the respective unstimulated control samples [11]. Regarding our data, the SRC pathway may be inherently downregulated in cSCC. Therefore, the SCR-inhibitory capacity of tirbanibulin described in other cell types [11, 12] does not seem to be the driving force regarding effects of tirbanibulin in the keratinocytic cell lines analyzed. We confirmed this via analyses of downstream SRC-targets, which were not altered following tirbanibulin stimulation. Potential effects on pSRC by tirbanibulin in keratinocytic cell lines with higher baseline pSRC levels are possible, however, were not evaluated in this study.

Next, we focused on tirbanibulin’s interference with microtubule formation: Polymerization of β-tubulin was significantly inhibited by tirbanibulin in A431, while in SCC-12 and especially NHEK, again, no alterations were detected. Tubulin’s isotypes, α- and β-tubulin, congregate to heterodimers which polymerize to microtubules. Microtubules are critical players in cell migration, protein transport, and mitosis by forming the mitotic spindle [18]. Thus, inhibition of tubulin-polymerization, e.g., by tirbanibulin, induces cell death via cell cycle arrest in the G2/M-phase resulting in apoptosis [12, 19]. These effects could be confirmed in A431 following tirbanibulin treatment.

The inhibitory effect of tirbanibulin on β-tubulin-polymerization was also observed in the cervix carcinoma cell line HeLa. Here, tirbanibulin induced cell cycle arrest via binding to the colchicine-binding site of β-tubulin. After washout of tirbanibulin, no residual toxicity was detected. Therefore, in contrast to other tubulin-targeting drugs, tirbanibulin’s reversible binding to tubulin may explain the comparatively low toxicity in clinical trials [13].

Since microtubules are also involved in cell migration [20], most observations following tirbanibulin treatment—reduced proliferation, reduced migration, induced apoptosis—may be mediated exclusively by dysregulation of β-tubulin-polymerization.

In a further approach, we analyzed effects of tirbanibulin on cytokine and chemokine secretion. We detected an increased secretion of VEGF-C, VEGF-D, IL-1α and bFGF, suggesting a tirbanibulin-mediated proinflammatory effect. These results confirm recent data, demonstrating an increase of proinflammatory cytokines in the keratinocyte cell line KERTr following tirbanibulin treatment, especially in comparison to other topical treatment options, e.g., 5-fluorouracil. Among the increased cytokines, IL-8 and IL-1α were the most prominent. Relative fold changes concerning the upregulation of IL-1α are comparable with our results, however, no absolute concentrations are given in the study by Schlesinger et al. [21]. The additional slight upregulation of IL-8 (1.5fold) by tirbanibulin could not be detected in our analyses [21].

IL-1α, a proinflammatory cytokine, plays a divergent role in tumorigenesis; pro- as well as anti-tumor effects are described: while IL-1α may support cell cycle arrest, T-cell activation, and tumor regression in some studies, tumor-promoting effects in other studies include induction of proliferation, angiogenesis, and lymph angiogenesis. The latter is also described for bFGF, next to aspects such as anti-apoptosis, immune evasion, and invasion [22].

VEGF-C and VEGF-D stimulate angiogenesis via binding to VEGF receptor 3 (Flt-4) [23], thereby promoting metastasis in murine tumor models including a carcinogen-induced cSCC tumor mouse model [24]. Interestingly, in early malignant skin lesions elevated VEGF-D levels were associated with tumor regression via anti-tumoral M1/Th1/Th17 polarization of immune cells in the microenvironment. Direct effects of VEGF-C or VEGF-D on tumor cells are not described [24]. In line with these observations, we detected an increase of VEGF-D in all analyzed cell lines, the most pronounced in healthy keratinocytes (NHEK), the least in the more dedifferentiated cSCC cell line (SCC-12).

Overall, we demonstrate that tirbanibulin inhibits proliferation and migration, as well as inducing apoptosis via G2/M-cell cycle arrest in cSCC cells lines. These effects are most likely mediated via dysregulation of β-tubulin-polymerization, and not via SRC-signaling. An additional effect, however, may be conveyed via proinflammatory and proangiogenic cytokine release in early cSCC cells. This may also partly explain the AEs of topical tirbanibulin treatment, such as local irritation including erythema.

Notably, across all analyses, NHEK were less influenced by tirbanibulin than cSCC cell lines, which can explain the overall good tolerability of the compound. Regarding the cSCC cell lines, especially A431 were affected by tirbanibulin stimulation. In general, SCC-12 are considered to be in a more dedifferentiated cellular state than A431, thus suggesting, that healthy keratinocytes as well as higher-grade cSCC cells are less susceptible to tirbanibulin, thereby underlining tirbanibulin’s effects as cancer specific phenomena [11].

## Data Availability

No datasets were generated or analysed during the current study.
